# A Novel Combined Conjugate Therapeutic Cancer Vaccine, Recombinant EGF-CRM197, in Patients With Advanced Solid Tumors: A Phase I Clinical Study

**DOI:** 10.3389/fonc.2021.745699

**Published:** 2021-11-05

**Authors:** An-Wen Xiong, Jue-Min Fang, Sheng-Xiang Ren, Wei Li, Jing Wang, Yu Zhao, Guo-You Chen, Qing Xu, Cai-Cun Zhou

**Affiliations:** ^1^ Department of Medical Oncology, Shanghai Pulmonary Hospital, Tongji University School of Medicine, Shanghai, China; ^2^ Shanghai Tenth People’s Hospital, Tenth People’s Hospital of Tongji University, Shanghai, China; ^3^ Shanghai Humantech Biotechnology Co., Ltd, Shanghai, China

**Keywords:** therapeutic cancer vaccine, EGF-CRM197, Solid tumors, clinical trial, immunotherapy

## Abstract

**Introduction:**

The therapeutic cancer vaccine recombinant Epidermal Growth Factor (EGF)-CRM197 is a novel combined conjugate EGF with CRM197 as a carrier protein. Immunization with the EGF-CRM197 vaccine can induce high levels of neutralizing anti-EGF antibodies that inhibit EGF/EGFR signaling and thereby suppress growth of tumors that rely on this signaling pathway. Herein, we characterize the humoral immune responses elicited by the recombinant EGF-CRM197 vaccine in patients with advanced solid tumors in a phase I clinical trial and assess the safety, tolerability, and immunogenicity of this vaccine (CTR20190473).

**Methods:**

A total of 16 subjects were enrolled in this study. Under 6 + 3 design, patients in each dosing cohort were administrated subcutaneously at a dosage of 0.4 mg, 0.8 mg, and 1.6 mg, respectively. The patients received vaccinations for immune induction (once a week for 4 consecutive weeks) and booster vaccinations (once every 4 weeks). Safety evaluation was performed 1 week after the immune induction. Booster vaccination was given until the occurrence of disease progression, intolerance, withdrawal of informed consent by the patient, or negative result of anti-EGF test after two booster vaccinations.

**Results:**

Vaccination with EGF-CRM197 is safe and well-tolerated in patients with advanced solid tumors. Adverse reactions at the injection site were the most common adverse events (AEs) in recipients. No severe adverse reactions post vaccination were observed in the present study. Vaccinated patients developed a robust neutralizing antibody response triggered by EGF-CRM197 that significantly reduced the levels of EGF in serum. For lung cancer patients who were super good antibody responders (sGAR) to EGF-CRM197, the median progress-free survival (PFS) was 4.83 months, significantly longer than that of the good antibody responder (GAR) patients with lung cancer whose median PFS was 2.10 months (P=0.0018). The median overall survival (OS) of GAR lung cancer patients was 10.67 months while the OS) for sGAR lung cancer patients was not reached until analysis was performed. The median follow-up of the sGAR lung cancer patients was 14.6 months.

**Conclusion:**

Our study demonstrates that the recombinant EGF-CRM197 therapeutic cancer vaccine can induce a good immune response in patients with advanced solid tumors and is safe and well tolerated, which ensures further clinical development of the vaccine for extending the survival time of EGF-CRM197 sensitive patients with advanced solid tumors.

**Clinical Trial Registration:**

http://www.chinadrugtrials.org.cn, identifier CTR20190473, EGF-CRM197.

## Introduction

Immunotherapy has emerged as a promising approach and a standard pillar in the treatment of cancer (1–3). In addition to immune checkpoint inhibitors and adoptive cell therapy, the therapeutic cancer vaccine has become an important new addition to cancer immunotherapy. In 2010, it was the approval of the therapeutic cancer vaccine Provenge that sparked an upsurge in tumor immunotherapy. To date, promising clinical results have been obtained from the application of a variety of therapeutic cancer vaccines in the treatment of many different types of tumors (4). The most common approach to cancer therapeutic vaccines uses tumor antigens to mount a specific immune response to tumor cells by activating cell-mediated immunity. However, few cancer vaccines function by eliciting humoral immune responses.

Current therapeutic antibody-based drugs such as cetuximab, panitumumab, and nivolumab comprise one of the predominant groups of drugs for the treatment of cancer. However, the price of these antibody drugs is concerning as the costs of the treatments continue to rise significantly. A recent survey (5) has shown that 42% of patients believe that their cancer treatment has caused a heavy or even catastrophic family economic burden. As a result, 20% seek out reduced treatment and 24% of patients end therapy completely. Furthermore, because of heterogeneity, complexity, development of drug resistance, and the sheer diversity of tumors, much efforts are needed for the discovery of effective therapeutic drugs with lower costs for the treatment of cancer (6, 7). The EGF-EGFR signaling pathway plays a pivotal role in tumorigenesis and progression of tumors (8). Studies have shown that EGFR is highly or aberrantly expressed in many solid tumors. EGFR is implicated in tumor cell proliferation, angiogenesis, tumor invasion, metastasis, and inhibition of tumor cell apoptosis. Therefore, a therapeutic strategy directed at inhibiting EGFR-mediated signaling pathways is a feasible therapeutic option in cancer patients.

The recombinant EGF-CRM197 tumor therapeutic vaccine is a novel vaccine developed by Shanghai Humantech Biotechnology Co., Ltd. The preclinical data including safety and efficacy of EGF-CRM197 vaccine have been submitted to the Center for Drug Evaluation (CDE) of China and approved for clinical trial by the CDE (Approval No.:2017L04930).

CRM197 in this vaccine is a diphtheria toxin mutant obtained from the strain of the virulence gene mutant strain of Bacillus diphtheriae, which has been widely used in a variety of commercial preventive vaccines for infectious diseases as a safe and efficient carrier protein, such as haemophilus influenzae type B vaccine (Wyeth, Novartis), 13-valent pneumococcal conjugate vaccine (Wyeth) and so on. EGF-CRM197 was developed using diphtheria toxin mutant CRM197 as a carrier protein to couple to EGF and Montanide ISA 51 VG as an adjuvant. The EGF-CRM197 vaccine is aimed to treat tumors characterized by high levels of EGF secreted from tumor cells, such as in a non-small cell lung cancer. Vaccination of EGF-CRM197 can induce the production of high levels of neutralizing antibodies against EGF, thereby inhibiting the EGF/EGFR signaling pathway and the growth of tumors relying on it. EGF-CRM197 has been shown to be immunogenic, well tolerated, and exhibit antitumor activity in previous preclinical studies.

Clinical applications of the CIMAvax-EGF vaccine, one that is similar to EGF-CRM197, has proved to be safe and effective. The CIMAvax-EGF vaccine shows strong immunogenicity, effectively neutralizes EGF, increases the titer of anti-EGF antibodies, and significantly prolongs the survival time of patients with high serum EGF levels (9, 10). In the present phase I clinical trial approved by the National Medical Products Administration (CTR20190473, http://www.chinadrugtrials.org.cn), the safety, dose-limiting toxicity, tolerability, and immunogenicity of the EGF-CRM197 vaccine were evaluated in patients with advanced solid tumors.

## Patients and Methods

### Study Design and Patients

This multicenter, non-randomized, open, dose-escalation phase I clinical trial was carried out in Shanghai Pulmonary Hospital and Shanghai Tenth People’s Hospital, Shanghai, China. A total of 16 patients with advanced solid tumors were enrolled. All enrolled patients were informed of the study and the potential risks. Written informed consent has been obtained from each patient prior to the study specific procedures. The clinical trial protocol, informed consent, and researcher brochure were approved by the ethics boards from the Medical Ethics Committee of Shanghai Pulmonary Hospital (Approval letter No.:18203ZL-1) and the Medical Ethics Committee of Shanghai Tenth People’s Hospital (Approval letter No.:SHSY-IEC-4.1/19-111/02) and by the National Regulatory Agency. The study was conducted in accordance with the principles of the Declaration of Helsinki and Good Clinical Practices guidelines.

Patients received the vaccine at a dose of 0.4 mg (cohort 1), 0.8 mg (cohort 2), and 1.6 mg (cohort 3). The study was performed in each cohort according to the 6 + 3 dose criteria. 2 cases of dose limiting toxicity (DLT) were observed in 6 patients in cohort 1-3 upon which 3 more patients were added to the cohort. For example, when 2 cases of DLT occurred in 6 patients or 3 cases of DLT in 9 patients (6 + 3), the next study would be started in cohort 1 to 3 after completing the safety evaluation of the immune induction; if more than 3 DLTs are observed in 9 (6 + 3) patients, the dose escalation will be terminated. If patients drop out due to non-drug safety reasons, new patients can be added until 9 (6 + 3) patients have completed the safety evaluation during induction, 1 week after the fourth administration.

9 (6 + 3) patients were planned to be enrolled in cohort 1. After the first patient completes the safety evaluation one week after the fourth dose, other patients of this cohort will be enrolled. When more than 3 DLTs are observed among 9 (6 + 3) patients in cohort 3, the study on cohort 4 will begin. After completing the study on cohort 4, the safe dosage will be determined based on the results. After completing the cohort 3 trial, if maximum tolerated dose (MTD) is not reached (i.e. more than 2 DLT cohort doses used in the previous cohort are observed), the subsequent increase in dosage will be determined based on the actual situation (cohort 5) and the outcome analysis of vaccine components conducted in cohort 3. When cohort 3 can’t be reached, an appropriate dose for the outcome analysis (3 cases) will be determined according to actual situation.

The occurrence of toxicity is classified according to the standard version 4.03 (CTCAE 4.03) of Common Terminology Criteria for Adverse Events. When the following toxicity occurs, it is defined as the DLT of EGF-CRM197 vaccine: ≥ Grade 4 hematological toxicity or ≥ Grade 3 non-hematological toxicity, and treatment-related serious adverse events (SAE). MTD is defined as the dose of DLT observed in greater than 33% of subjects below 1 dose level.

Health status assessment of the patients included physical examination (vital signs, weight, PS score, skin at the injection site, etc.), a hematologic and biochemical profile, a routine urine and stool test, and an electrocardiogram performed before and after vaccination. Tumor assessment by CT/MRI scan was performed 50 ± 3 days (once every 8 weeks) post vaccination and the clinical efficacy evaluated according to Response Evaluation Criteria in Solid Tumors version 1.1 (RECIST1.1). Categories of the evaluation of the response to treatment included complete response (CR), partial response (PR), stable disease (SD), and progressive disease (PD). The disease control rate (DCR) is defined as CR+PR+SD. PFS is the length of time from random assignment in a clinical trial to the first recorded disease progression or death from any cause. The overall survival time is the time from the signing of the informed consent to the death from any cause of the subject.

To examine the concentrations of EGF and anti-EGF antibody titer in serum, blood samples were collected on day 1(prior to immunization), 15, 29, 50, 64, and 78 post immunization with EGF-CRM197. An enzyme-linked immunosorbent assay (ELISA) kit (Quantikine EGF, R&D Systems Inc, Cat: DEG00) was utilized according to the manufacturer’s instructions. The ELISA was performed by Frontagelab, Shanghai, China.

Patients enrolled were aged 18 to 70 years, regardless of gender, and diagnosed histologically or cytologically with an advanced inoperable neoplastic lesion (RECIST, 1.1). For these patients, prior standard regimens (platinum-based therapy, chemotherapy, targeted therapy or immunotherapy) have failed and there have been no further treatment within four weeks. The expected survival of these patients was no less than 3 months (with a PS score of 0 to 1. For female subjects, the result of pregnancy tests was negative and there was no plan of becoming pregnant in six months.

Excluded from this study were patients who received immunosuppressant therapy within 1 month prior to the start of the study, patients who received any immunotherapy within 3 months, and those who received oral, intramuscular or intravenous corticosteroids within the last 30 days. However, patients with a single oral, intramuscular or intravenous injection of dexamethasone (≤ 5 mg) or other hormonal therapies with equivalent efficacy 14 days prior to the first dose of the vaccine and patients taking inhaled corticosteroids for the treatment of respiratory insufficiency syndrome or treated with topical steroids were qualified for the present study. Patients with the following diseases or history were excluded from the study: uncontrollable epilepsy; mental and neurological disorders causing cognitive impairment; a history of alcohol or drug abuse within 6 months; a history of severe allergic reactions or allergic constitution; splenectomy; autoimmune diseases or immunodeficiency; secondary malignant neoplasm within 5 years except for basal cell carcinoma or cervical intraepithelial neoplasia; systemic diseases such as active infection, cirrhosis, chronic kidney disease, severe chronic lung disease, hypertension, unstable angina, congestive heart failure, or myocardial infarction within 1 year; bone marrow disorders (hemoglobin<90 g/L, WBC<3×10^9^/L, platelets<75×10^9^/L); abnormal liver and kidney function [total bilirubin (TBIL) > 1.5 times the upper limit of normal (ULN); aspartate aminotransferase (AST) and alanine aminotransferase (ALT)>2.5 times ULN or > 5 times ULN (for liver metastases); serum creatinine> 1.5 ULN].

### Administration of EGF-CRM197 Vaccine

The EGF-CRM197 vaccine (Shanghai Humantech Biotech, China; batch number: 20180330, 20200101) was emulsified with an equal volume of the adjuvant Montanide ISA 51 VG (Shanghai Humantech Biotech, China; batch number: 20180515, 20200101). No greater than 1ml of the emulsified vaccine was injected subcutaneously at two sites: lower edge of the deltoid muscle in the upper arm or the lower edge of the gluteus maximus.

Patients in each cohort received four injections of the vaccine at day 1 ± 1, 8 ± 1, 15 ± 1, and 22 ± 1 day for immune induction. Booster vaccination was initiated in each cohort 4 weeks after the fourth vaccination (50 ± 3 days). The vaccine was injected once every 4 weeks until the occurrence of disease progression, intolerance, withdrawal of informed consent by the patient, or negative result of anti-EGF test after two booster vaccinations.

### Measurement of the Concentrations of EGF and CRM197 in Serum

To determine the concentrations of EGF and CRM197 in serum, 5 ml of blood samples from the subjects were collected before the first vaccine injection and then 0.5h ± 5min, 1h ± 10min, 2h ± 10min, 4h ± 20min, 8h ± 30min, 24h ± 60min, 48h ± 60min, 96h ± 6h, and 144h ± 6h post first vaccination. ELISA was performed by Frontagelab using ELISA kits (Quantikine EGF, R&D Systems Inc, Cat: DEG00; Quantikine Diphtheria Toxoid, R&D Systems Inc, Cat:940-DTX-AG1); according to the manufacturer’s instruction.

### Statistical Analysis

Descriptive statistical analysis was used for the safety evaluation of EGF-CRM197 and the measurement (or analysis) of the levels of EGF and anti-EGF antibody. OS and PFS were analyzed using the Kaplan–Meier method. Log-rank test was used for statistical comparisons between survival curves. Statistical analysis was performed using the GraphPad Prism 7.0 software (InStat, GraphPad Software). A two-sided P value of less than 0.05 was considered significant.

## Results

### The CONSORT Flow Diagram

**Figure 1:**
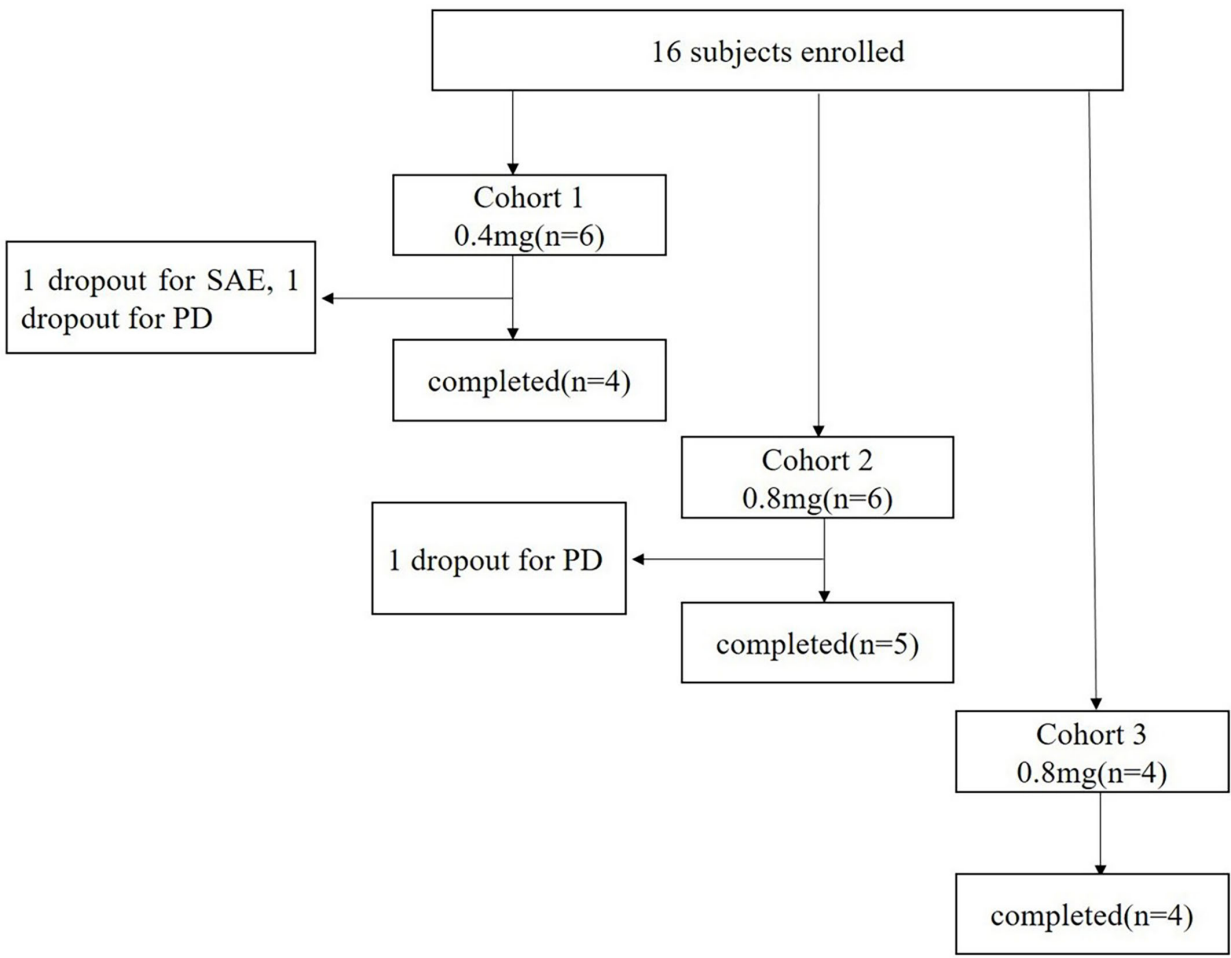
CONSORT Flow Diagram.

A total of 16 patients with advanced solid tumors were enrolled from April 10, 2019 to July 15, 2020, and assigned to three different groups treated with 0.4 mg, 0.8 mg, and 1.6 mg of EGF-CRM197, respectively (**Figure 1**). Completion of the study means that the patient received 4 immunizations for immune induction and a safety assessment of the vaccine one week after the 4th immunization on day 29 post first vaccination.

### Patient Characteristics (or Demographic Information)

Among the 16 patients in the present study, 12 were male and 4 were female with a median age of 59.54 years (43.46-69.59 years). The average height was 167.50 ± 8.60cm (155.00-185.00) cm. The average weight was 65.65 ± 13.31 (56.00-75.95) kg. There was 1 case with a PS score of 0 points (6.25%) and 15 cases with a PS score of 1 point (93.75%). There were 13 cases of lung cancer, 2 cases of colorectal cancer, and 1 case of pancreatic cancer. The patient’s EGFR and KRAS mutations were not tested. The patient characteristics are shown in **Table 1**.

**Table 1:** Baseline characteristics.

Characteristics	Allocation		
	Cohort1 (0.4mg)	Cohort 2 (0.8mg)	Cohort 3 (1.6mg)
**Age**, yr	64.3	57.6	62.55
**Gender**, n (%)			
Male	4 (66.67%)	4 (66.67%)	4 (100.00%)
Female	2 (33.33%)	2 (33.33%)	0 (0.00%)
**Height** (cm), Ave (SD)	162.67 (5.24)	172.67 (10.71)	167.00 (5.72)
**Weight** (kg), Ave (SD)	62.17 (7.25)	73.62 (15.82)	58.93 (13.01)
**PS core**, n (%)			
0 point	0 (0.00%)	1 (16.67%)	0 (0.00%)
1 point	6 (100.00%)	5 (83.33%)	4 (100.00%)
**Type of cancer**, n (%)			
Lung cancer	6 (100.00%)	6 (100.00%)	1 (25.00%)
Squamous cell lung cancer	3 (50.00%)	1 (16.67%)	0 (0.00%)
Lung adenocarcinoma	3 (50.00%)	5 (83.33%)	0 (0.00%)
Unknown	0 (0.00%)	0 (0.00%)	1 (25.00%)
Colorectal cancer	0 (0.00%)	0 (0.00%)	2 (50.00%)
Pancreatic cancer	0 (0.00%)	0 (0.00%)	1 (25.00%)

Yr, year; Ave, average; SD, standard deviation.

There were 6 patients in the 0.4 mg and 0.8 mg groups, respectively, and 4 patients in the 1.6 mg group. 5 patients (83.33%) in the 0.4 mg group completed 4 immunizations and 2 patients (33.33%) received booster immunizations. 5 patients in the 0.8 mg group (83.33%) completed 4 immunizations and 3 patients (50%) received booster immunizations. 4 patients (100%) in the 1.6 mg group completed 4 immunizations and 1 patient (25%) received booster immunizations.

### Safety Assessment of EGF-CRM197 Vaccine

Safety assessment of EGF-CRM197 vaccine was carried out on all of the subjects who received at least one vaccination with EGF-CRM197. According to CTCAE 4.03, DLT is any grade 3 non-hematological toxicity or grade 4 hematological toxicity that is possibly related to the treatment. MTD is defined as the dose level below which more than 33% of subjects experience a DLT. DLTs at each dose level in this study were collected to determine the primary endpoint MTD. No DLT was observed in all of the subjects receiving EGF-CRM197 treatment. One subject in the 0.4 mg group had SAE, but it was not related to the treatment. There was one subject in the 0.4 mg group and one in the 0.8 mg group that dropped out of the study due to PD.

The safety of the vaccine was determined by the total number of patients who experienced any serious and non-serious AE reported and assessed through systematic evaluation using the terms in CTCAE 4.03. Among all subjects in the 3 cohorts, 11 cases (68.75%, 46 times) reported AE related to the treatment that were graded I to II (36 cases of grade I and 10 cases of grade II). No treatment-related AE above grade 3 was observed. No subjects withdrew from the study due to treatment-related AEs. There was no significant difference in the incidence of AEs related to treatment and severity between the groups. The summary of adverse events is shown in **Table 2**.

**Table 2:** EGF-CRM197 treatment -related AEs.

Items	0.4 mg group (n = 6)		0.8 mg group (n = 6)		1.6 mg group (n = 4)	
	Cases	Patients	Cases	Patients	Cases	Patients
Loss of appetite	0	0	0	0	1	1
Grade II	0	0	0	0	1	1
An elevation of γ-glutamyltransferase	0	0	0	0	1	1
Grade I	0	0	0	0	1	1
A low white blood cell count	0	0	0	0	1	1
Grade I	0	0	0	0	1	1
An elevation of alanine aminotransferase	2	2	0	0	0	0
Grade I	2	2	0	0	0	0
A low red blood cell count	1	1	0	0	0	0
Grade II	1	1	0	0	0	0
Increase in body temperature	0	0	0	0	1	1
Grade I	0	0	0	0	1	1
An elevation of aspartate aminotransferase	4	3	0	0	0	0
Grade I	4	3	0	0	0	0
Elevated bilirubin	0	0	2	1	0	0
Grade I	0	0	2	1	0	0
Elevated unconjugated bilirubin	0	0	1	1	0	0
Grade I	0	0	1	1	0	0
Low hemoglobin	1	1	0	0	0	0
Grade II	1	1	0	0	0	0
An elevation of creatinine	1	1	0	0	0	0
Grade I	1	1	0	0	0	0
An elevation of alkaline phosphatase	0	0	0	0	1	1
Grade I	0	0	0	0	1	1
hyperglycemia	0	0	2	2	0	0
Grade I	0	0	2	2	0	0
An elevation of blood lactate dehydrogenase	0	0	0	0	1	1
Grade I	0	0	0	0	1	1
Thrombocytopenia	0	0	0	0	3	2
Grade I	0	0	0	0	3	2
Dizziness	1	1	0	0	0	0
Grade I	1	1	0	0	0	0
Rash	1	1	0	0	0	0
Grade I	1	1	0	0	0	0
Fever	1	1	0	0	0	0
Grade II	1	1	0	0	0	0
Erythema at vaccine injection site	1	1	0	0	0	0
Grade I	1	1	0	0	0	0
Reactions at vaccine injection site	0	0	1	1	4	2
Grade II	0	0	1	1	1	1
Grade I	0	0	0	0	3	2
Pain at vaccine injection site	0	0	0	0	2	1
Grade I	0	0	0	0	2	1
Induration at vaccine injection site	1	1	3	2	5	3
Grade II	0	0	1	1	1	1
Grade I	1	1	2	2	4	3
Constipation	1	1	0	0	0	0
Grade I	1	1	0	0	0	0
Diarrhea	0	0	0	0	1	1
Grade II	0	0	0	0	1	1
Lower abdominal pain	0	0	0	0	1	1
Grade II	0	0	0	0	1	1
Total	12	5	9	3	22	3

The treatment-related AEs observed in this trial were mainly adverse reactions at the injection site, including induration, erythema, and tenderness. 2 subjects (3 times) in the 0.4 mg group (33.33%), 2 subjects (4 times) in the 0.8 mg group (33.33%), and 3 subjects (11 times) in the 1.6 mg group (75%) experienced local injection site adverse reactions (grade I-II AE) that were mild and only symptomatic treatment was needed. This result suggests that the EGF-CRM197 vaccine is safe and well tolerated in patients with advanced solid tumors.

A total of 6 subjects (20.69%) among 29 patients in this study reported SAE. Among those patients, 2 excluded from the study had 2 SAEs in the screening phase. 6 SAEs were reported in 4 patients enrolled in the groups with treatment, including 1 patient with coronary artery disease, 1 patient with tracheoesophageal fistula, 1 patient with edema in the lower extremities, and 1 patient with left shoulder pain. Among them, 1 patient died of hemoptysis caused by tracheoesophageal fistula. These SAEs had been evaluated to be definitely not or probably not related to treatment with EGF-CRM197.

### Production of High Levels of Anti-EGF Antibody and Neutralization of EGF Induced by EGF-CRM197 Vaccination in Patients With Advanced Solid Tumors

**Figure 2:**
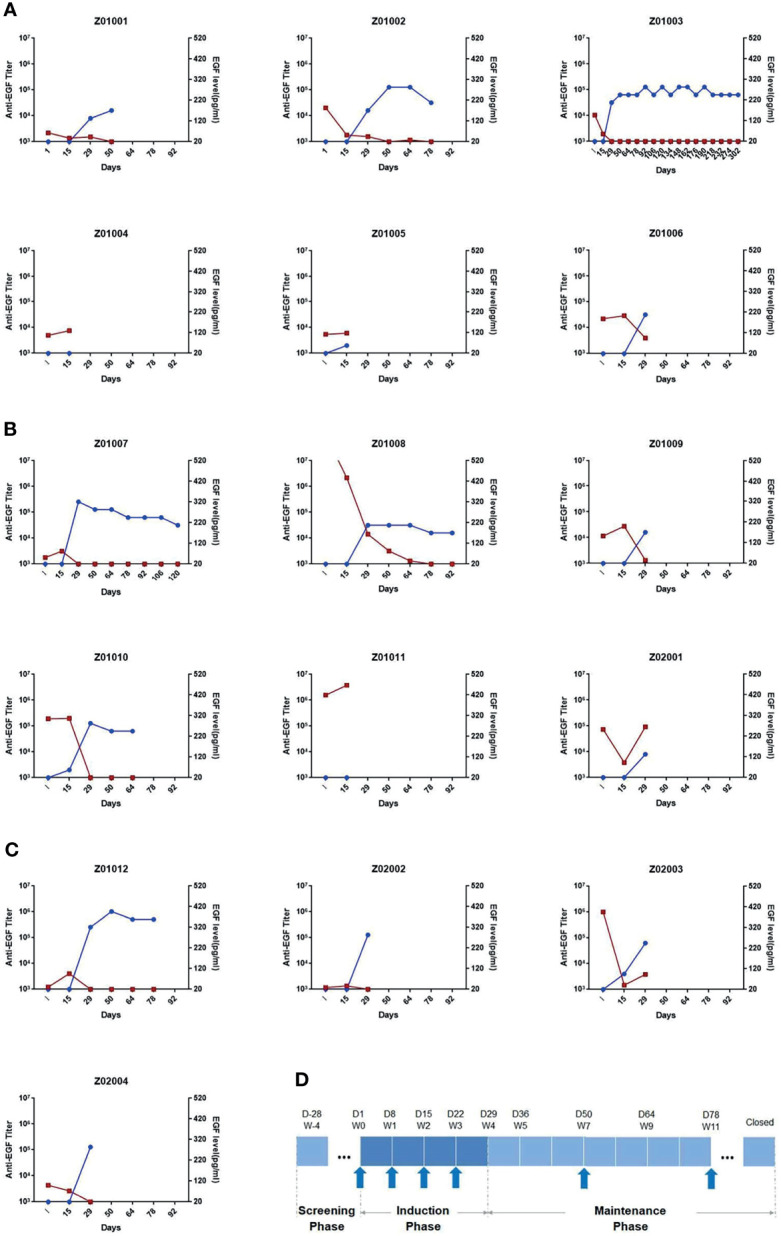
The titers of anti-EGF antibody and the levels of EGF in serum in each subject receiving EGF- CRM197 treatment of 0.4mg **(A)**, 0.8mg **(B)** and 1.6mg **(C)**. (Blue: titers of anti-EGF antibody; Red: the levels of EGF in serum). **(D)** Study workflow and profile of EGF-CRM197 vaccine administration.

The immune responses to EGF-CRM197 vaccination in patients with advanced solid tumors were evaluated by measuring anti-EGF antibody titer and the concentration of EGF in serum. Blood samples were collected prior to immunization (day 1), and at 15, 29, 50, 64, and 78- days post EGF-CRM197 administration, when an ELISA assay was performed. The correlation between the titers of anti-EGF antibody and serum EGF levels in each subject was analyzed. As shown in **Figure 2**, 4 immunizations with EGF-CRM197 for immune induction could successfully induce the production of high levels of anti-EGF antibodies that effectively neutralized EGF in these patients. Serum EGF dropped to an undetectable level in 12 out of 13 patients (92.31%). The levels of anti-EGF antibody remained at a high level for long periods of time after booster immunization (once every 4 weeks).

### The Assessment of Short-Term Efficacy of EGF- CRM197

The assessment of short-term efficacy of EGF- CRM197 on CR, PR, SD, PD, response rate (CR + PR), and disease control rate (CR + PR + SD) was performed on patients who had completed the 4 vaccinations for induction and booster vaccinations. The PD was the endpoint event for the evaluation of overall efficacy and survival analysis was performed based on the data of overall efficacy. Survival analysis of overall efficacy data was performed separately for the subgroups of lung cancer subjects. Given that all of the subjects enrolled in this study were at least in the third-line setting and standard cancer treatments had failed to improve the subjects’ outcome, and that it takes nearly 4 weeks for the vaccination to be effective, there was no significant improvement made in the treatment outcome in terms of the response rate (CR+PR) and the disease control rate (CR+PR+SD).

**Figure 3:**
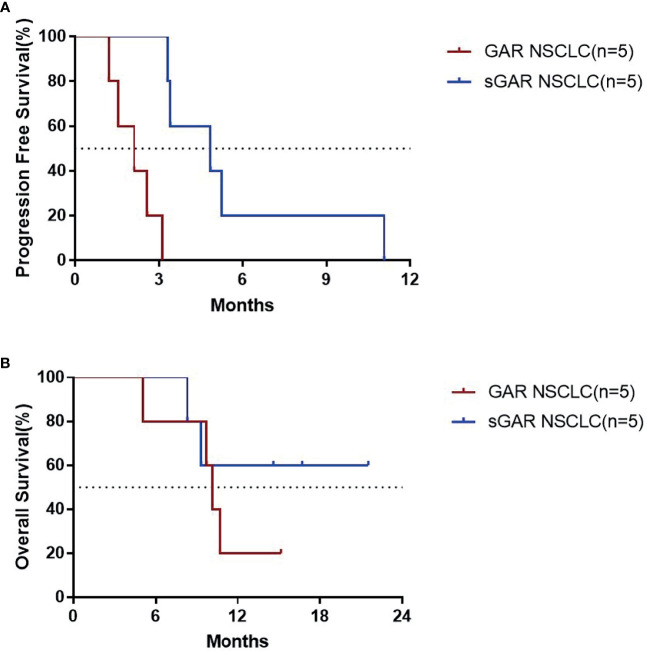
PFS **(A)** and OS **(B)** for the patients with lung cancer who completed the 4 vaccinations for immune induction.

Patients were classified as good antibody responders (GAR) if they elicited an antibody titer equal or higher than 1:4000, poor antibody responders (PAR) if the titers were lower than 1:4000, and super good antibody responders (sGAR) if the elicited antibody titers were equal or higher than 1: 64000 (11). We found that in lung cancer patients who were sGAR (n=5), the median PFS was 4.83 months, which was significantly longer than that of GAR lung cancer patients (n=5, median PFS was 2.10 months; P=0.0018). The median OS for GAR lung cancer patients was 10.67 months and had not been reached in sGAR patients until the analysis was performed. The median follow-up time for sGAR lung cancer patients was 14.6 months (**Figure 3**). Our results are consistent with the previous findings from clinical trials for CIMAvax EGF vaccine.

### Changes in the Levels of EGF- CRM197 Vaccine Components in Patients

The EGF- CRM197 vaccine is mainly composed of human EGF protein and carrier protein CRM197. 3 subjects (75%) in the 1.6 mg group underwent outcome analysis of vaccine components.

The levels of CRM197 increased within 8 hours post initial vaccination in the 3 subjects, were undetectable in 2 patients after 24 h, and reached 1.33 ng/ml (limit of detection: 1.250 ng/ml) at 144 h in the rest of the patients. 3 subjects (100%) showed a transient increase in serum EGF levels that gradually decreased to 100 pg/ml within 8 h. The EGF levels in 1 subject increased progressively to 200 -300 pg/ml within 144 h but remained below 100 pg/ml in the other 2 patients. The influence of endogenous EGF in serum also needs to be considered in this analysis. The serum EGF in all of the 3 subjects dropped to undetectable levels after 4 immunizations for immune induction.

## Discussion

Despite advances in treatment, cancer remains one of the leading causes of death and economic burden worldwide. The overall cure and survival rates for advanced solid tumors remain very low. Therefore, it is urgent to develop new therapeutic drugs with expanding clinical benefit at lower costs than current treatments to improve the outcome of patients with cancer. Remarkable advances in antibody-based drugs, cell therapy, and immune checkpoint inhibitors have been achieved in cancer immunotherapy in recent years. A therapeutic vaccine based on the induction of antitumor immune responses to tumor-associated or specific target antigens has emerged as an attractive approach for the treatment of cancer. To date, induction of cellular immune responses underlies the therapeutic effect of most of the cancer vaccines.

The recombinant EGF-CRM197 cancer vaccine exerts its antitumor effect by inducing the production of neutralizing antibodies against serum EGF. EGF in serum can bind to and activate EGF receptors, thereby repressing cell apoptosis, stimulating cell proliferation, and inducing malignant transformation, angiogenesis, and metastasis. Studies have shown that serum EGF levels are correlated with tumorigenesis and prognosis of cancer patients. In a phase III clinical study, it was found that, at a cutoff value of 870 pg/mL, the survival rate of NSCLC patients with high EGF levels was significantly lower than that of patients with low EGF levels (HR, 0.38; 95% CI, 0.20-0.70; P=0.002). The median OS of the high EGF group was 8.63 months (95% CI, 1.15-28.28), while the median OS of the low EGF group was 15.06 months (95% CI, 1.67-15.59). These findings suggest that high levels of serum EGF are associated with the poor prognosis of NSCLC patients (10). Studies have also shown that the levels of serum EGF were elevated in various types of cancer, such as lung, colorectal, esophageal, liver, and ovarian cancers (12–15). In our previous study, the concentrations of EGF were examined in serum samples from 154 healthy donors and 57 patients with lung cancer. The levels of EGF in serum of lung cancer patients ranged from 344 pg/ml to1586 pg/ml, the median concentration was 788 pg/ml, and the average concentration was 923 pg/ml, all of which were significantly higher than that of healthy donors (257 ± 168 pg/ml) (data not published). A variety of targeted therapies that block the EGF/EGFR pathway have proven to be an effective therapy for cancer. In a phase III clinical study (10), the CIMAVax-EGF vaccine was administrated to induce the production of anti-EGF neutralizing antibodies in patients with high serum levels of EGF. The CIMAVax-EGF vaccine treatment significantly increased the median OS from 8.63 months (95% CI, 1.15-28.28) to 14.66 months (95% CI, 8.34-20.98) and the five-year survival rate from 0% to 23%.

In the recombinant EGF-CRM197 vaccine, human EGF protein is coupled with diphtheria toxin mutant CRM197. To enhance immunogenicity and the titer of the anti-EGF antibodies induced, the vaccine was emulsified in Montanide ISA51 VG adjuvant (16). Recombinant EGF-CRM197 can induce the production of high levels of anti-EGF neutralizing antibodies in patients that inhibit the EGF/EGFR signaling pathway, thereby potentially suppressing the growth of tumors that rely on this signaling pathway.

Preclinical studies demonstrated the high immunogenicity, good tolerability, and antitumor activity of the recombinant EGF-CRM197 vaccine. In the present clinical trial, the recombinant EGF-CRM197 vaccine also demonstrated good safety and tolerability. No subjects withdrew from the study due to treatment-related AEs. There was no significant difference in the incidence of AEs related to the treatment and the severity of AEs between the groups. There was no DLT in each group in this study. Reported common treatment-related AEs of the CIMAvax-EGF vaccine, very similar to EGF-CRM197, are mainly grade 1 and grade 2 AEs, including fever, cold, shivering, nausea, vomiting, headache, injection site pain, skin rashes, and joint pain. In the present study, the subjects did not experience fever, vomiting, headaches or other high grade adverse reactions. The most grade 1-2 treatment-related AEs were adverse reactions at the local injection site, manifested by induration, erythema, and tenderness, which might be caused by the administration of Montanide ISA51 VG adjuvant.

In order to improve the efficacy of the vaccine, the EGF-CRM197 vaccine was formulated in water-in-oil emulsions (17). The treatment-related AEs were mild, could be relieved within 2 weeks, and did not influence the course of the study. No patients in the present study experienced SAE or died of EGF-CRM197 treatment. An early termination of enrollment in cohort 3 was prompted based on the profile of good safety and toleration of EGF-CRM197.

Unlike more tradition approaches, the main effector of EGF-CRM197is the humoral immune response. Thus, the production of specific antibodies can reflect dynamic changes in patients receiving this vaccine. In this study, the analysis of the immune response triggered by EGF-CRM197 showed that all 13 subjects who completed 4 immunizations for induction were GAR with significantly increased titers of anti-EGF antibodies in serum. Among those patients, 2 (50%) in the 0.4 mg group, 2 patients (40%) in the 0.8 mg group, and 4 patients (100%) in the 1.6 mg group were sGAR, demonstrating a dramatic decrease in levels of EGF in serum. EGF in serum decreased to undetectable levels in 12 out of 13 patients (92.31%) in the PPS set. In addition, the titers of anti-EGF antibody after 4 immunizations were positively correlated with the dose of EGF-CRM197, as evidenced by the highest anti-EGF antibody titer in the 1.6 mg group. Booster immunization (once every 4 weeks) can maintain high levels of antibody titers and significantly reduce the levels of EGF in serum. What’s worth mentioning, the dosage and frequency of use were significantly lower than those of antibody drugs (usually hundreds of milligrams per injection every 1-3 weeks), which might lighten the financial burden, reduce the frequency of administration and improve patient compliance. Unfortunately, an increase in response rate (CR+PR) and disease control rate (CR+PR+SD) has not been observed in this study. Our results indicate that the increase in antibody titer and decrease in the levels of EGF induced by the administration of EGF-CRM197 may be implicated in the clinical benefit seen in these patients. The median PFS of sGAR patients with lung cancer was significantly longer than that of GAR patients with lung cancer. The median OS was 10.67 months for GAR patients, whereas the median OS for sGAR patients has not been reached at the time of analysis. Despite the small sample size and the potential differences in patients between the groups, our findings suggest that the high levels of anti-EGF antibody titers induced by EGF-CRM197 may be directly related to its clinical benefit. The relationship between anti-EGF antibody titer and the efficacy of EGF-CRM197 will be further validated in future clinical studies.

Other clinical studies suggest that the high levels of antibody in sGAR may be related to the improved OS for patients. The results of clinical trials for the CIMAvax EGF vaccine showed that compared with PAR patients, the median survival time of GAR patients was significantly prolonged (11.87 *vs.* 7.7 months, P=0.0095) (18). In another clinical trial, the median survival time of 20 NSCLC patients was 18.74 months, and the survival time of sGAR patients was significantly longer than that of GAR patients (median survival time was 19.3 *vs.* 8.4 months, P=0.0036) (19). Therefore, the level of induced neutralizing antibodies may be an important indicator for evaluating the clinical efficacy of therapeutic vaccines. The EGF-CRM197 vaccine exhibited high immunogenicity by eliciting the production of very high levels of anti-EGF neutralizing antibodies at a 1.6 mg immunization dose.

## Conclusion

The recombinant EGF-CRM197 cancer therapeutic vaccine is safe, well tolerated, and can effectively induce the production of high levels of anti-EGF antibodies that neutralize EGF in patients with advanced solid tumors. Patients with high levels of anti-EGF antibody titers induced by EGF-CRM197 show significantly improved OS. In addition, the components of the vaccine were undetectable in the serum of these patients 29 days post immunization. Treatment with EGF-CRM197 could be a novel, safe, and effective therapeutic regimen for therapy against advanced solid tumors.

## Data Availability Statement

The original contributions presented in the study are included in the article/supplementary material. Further inquiries can be directed to the corresponding author.

## Ethics Statement

The studies involving human participants were reviewed and approved by Medical Ethics Committee of Shanghai Pulmonary Hospital (Approval letter No.:18203ZL-1) Shanghai Tenth People’s Hospital (Approval letter No.:SHSY-IEC-4.1/19-111/02). The patients/participants provided their written informed consent to participate in this study.

## Author Contributions

All authors had full access to all the data of this *post hoc* analysis. A-WX and the statistical team from Clinical Research Institute of Peking University performed the statistical analysis and A-WX provided medical writing services. All authors contributed to the article and approved the submitted version.

## Funding

This clinical study was sponsored by Shanghai Humantech Biotechnology Co., Ltd, China.

## Conflict of Interest

Author G-YC is employed by Shanghai Humantech Biotechnology Co., Ltd.

The remaining authors declare that the research was conducted in the absence of any commercial or financial relationships that could be construed as a potential conflict of interest.

This study received funding from Shanghai Humantech Biotechnology Co., Ltd. The funder had the following involvement: study design, data analysis and preparation of the manuscript.

## Publisher’s Note

All claims expressed in this article are solely those of the authors and do not necessarily represent those of their affiliated organizations, or those of the publisher, the editors and the reviewers. Any product that may be evaluated in this article, or claim that may be made by its manufacturer, is not guaranteed or endorsed by the publisher.
